# Higher body mass index is associated with worse hippocampal vasoreactivity to carbon dioxide

**DOI:** 10.3389/fnagi.2022.948470

**Published:** 2022-09-07

**Authors:** Lidia Glodzik, Henry Rusinek, Tracy Butler, Yi Li, Pippa Storey, Elizabeth Sweeney, Ricardo S. Osorio, Adrienne Biskaduros, Emily Tanzi, Patrick Harvey, Christopher Woldstad, Thomas Maloney, Mony J. de Leon

**Affiliations:** ^1^Department of Radiology, Brain Health Imaging Institute, Weill Cornell Medicine, New York, NY, United States; ^2^Department of Radiology, New York University Grossman School of Medicine, New York, NY, United States; ^3^Department of Biostatistics and Epidemiology, University of Pennsylvania, Philadelphia, PA, United States; ^4^Department of Psychiatry, New York University Grossman School of Medicine, New York, NY, United States

**Keywords:** Alzheimer’s disease, hippocampus, obesity, MRI, cerebral blood blow, cerebrovascular reactivity (CVR)

## Abstract

**Background and objectives:**

Obesity is a risk factor for cognitive decline. Probable mechanisms involve inflammation and cerebrovascular dysfunction, leading to diminished cerebral blood flow (CBF) and cerebrovascular reactivity (CVR). The hippocampus, crucially involved in memory processing and thus relevant to many types of dementia, poses a challenge in studies of perfusion and CVR, due to its location, small size, and complex shape. We examined the relationships between body mass index (BMI) and hippocampal resting CBF and CVR to carbon dioxide (CVR_CO2_) in a group of cognitively normal middle-aged and older adults.

**Methods:**

Our study was a retrospective analysis of prospectively collected data. Subjects were enrolled for studies assessing the role of hippocampal hemodynamics as a biomarker for AD among cognitively healthy elderly individuals (age > 50). Participants without cognitive impairment, stroke, and active substance abuse were recruited between January 2008 and November 2017 at the NYU Grossman School of Medicine, former Center for Brain Health. All subjects underwent medical, psychiatric, and neurological assessments, blood tests, and MRI examinations. To estimate CVR, we increased their carbon dioxide levels using a rebreathing protocol. Relationships between BMI and brain measures were tested using linear regression.

**Results:**

Our group (*n* = 331) consisted of 60.4% women (age 68.8 ± 7.5 years; education 16.8 ± 2.2 years) and 39.6% men (age 70.4 ± 6.4 years; education 16.9 ± 2.4 years). Approximately 22% of them (*n* = 73) were obese. BMI was inversely associated with CVR_CO2_ (β = −0.12, unstandardized B = −0.06, 95% CI −0.11, −0.004). A similar relationship was observed after excluding subjects with diabetes and insulin resistance (β = −0.15, unstandardized B = −0.08, 95% CI −0.16, −0.000). In the entire group, BMI was more strongly related to hippocampal CVR_CO2_ in women (β = −0.20, unstandardized B = −0.08, 95% CI −0.13, −0.02).

**Discussion:**

These findings lend support to the notion that obesity is a risk factor for hippocampal hemodynamic impairment and suggest targeting obesity as an important prevention strategy. Prospective studies assessing the effects of weight loss on brain hemodynamic measures and inflammation are warranted.

## Introduction

Over the past 2 decades, the prevalence of obesity in the United States has increased from 30 to over 40% (CDC, 2022). It is a recognized risk factor for cognitive decline and Alzheimer’s disease (AD)(Whitmer et al., 2005; Nguyen et al., 2014; Alford et al., 2018; Singh-Manoux et al., 2018); however, the mechanisms for this association are not fully explained.

Excessive body weight facilitates endothelial dysfunction and atherosclerosis through increased lipid storage (Rocha and Libby, 2009) and chronic low-grade inflammation (MacEneaney et al., 2010; Lumeng and Saltiel, 2011). Under normal physiological conditions, vasodilation (and vasoconstriction) is regulated by the secretion of factors derived, among others, from endothelial cells (Shepherd and Katusić, 1991). Endothelial impairment contributes to increased cerebrovascular resistance, diminished cerebral blood flow (CBF) (Coucha et al., 2018), and reduced cerebrovascular reactivity (CVR) (Wenzel et al., 2020).

Animal models of obesity showed that increased body weight is associated with the increased cerebrovascular myogenic tone (Osmond et al., 2009), impaired neurovascular coupling, and endothelium-dependent relaxation (Li et al., 2013), as well as alterations in wall structure and lumen of cerebral vessels (Osmond et al., 2009). All these factors are important determinants of cerebral vasodilation.

An important type of CVR, denoted CVR_CO2_, is the brain blood vessel response to changing levels of carbon dioxide (CO_2_). This important regulatory mechanism increases CBF in response to rising CO_2_ level, which thus increases H+ and constricts vessels when CO_2_ decreases. CVR_CO2_, and especially hypercapnic challenge, is considered a valuable marker of vascular health (Liu et al., 2019). CVR_CO2_ assessment has a diagnostic value in many vascular conditions, revealing diminished reserve, which otherwise would have not been detected with baseline CBF (Liu et al., 2019). Similarly, CVR_CO2_ evaluation is conducted in the context of dementia and cognitive impairment with the premise that functional abnormalities will be found earlier than the structural ones. Indeed, impaired CVR_CO2_ coincides with and predicts cognitive decline (Wolters et al., 2016; Alwatban et al., 2019; van der Thiel et al., 2019). Although there are ample bodies of literature showing relationships between CVR_CO2_ impairment and cognitive status (Glodzik et al., 2013; Wolters et al., 2016; Alwatban et al., 2019; van der Thiel et al., 2019; Kim et al., 2021), studies are mostly focused on global or regional neocortical metrics.

Hippocampal pathology is one of the earliest signs of AD (Glodzik-Sobanska et al., 2005) and is implicated in memory impairment; however, the structure itself is a challenging target for CVR_CO2_ studies due to technical difficulties related to its small size and location near vessels. The vascular network supplying the hippocampus differs from the architecture of cortical vessels. Hippocampal vasculature exhibits a relatively small number of cross-capillary connectors (anastomoses) and collateral vessels. As a result, the hippocampus has long been suspected of being highly vulnerable, having limited ability to cope with hypoperfusion (Ravens et al., 1968; Duvernoy, 2005). It may be even more susceptible in states of higher demand, such as those occurring during different types of physiological challenges. Less is known regarding how obesity affects hippocampal CVR_CO2_. In this study, we hypothesized that higher body mass index would be associated with worse vasoreactivity to CO_2_ in the hippocampus, consistent with (1) its vulnerability to hypoperfusion, (2) its central role in dementia pathology, and (3) known associations between BMI and dementia. Since both diabetes (Last et al., 2007) and insulin resistance (Frosch et al., 2017), linked to a higher BMI, are associated with impaired gray matter CVR_CO2_, to evaluate whether high body mass alone is related to altered CVR_CO2_, we also examined whether the relationships can be detected in subjects without diabetes and insulin resistance.

Women are at a greater risk of both dementia (Seshadri et al., 1997) and obesity (Chooi et al., 2019). Moreover, obesity (Whitmer et al., 2005) and measures of fat deposition (Ma et al., 2020) are more strongly associated with dementia in women than in men. Finally, hemodynamic response to CO_2_ challenge differs between sexes, with some authors showing greater vasoreactivity in women than in men (Kastrup et al., 1997; Minhas et al., 2018). Since it remains unknown whether excessive body weight affects CVR_CO2_ differently based on sex, we assessed BMI—CVR_CO2_ association separately in men and women.

## Materials and methods

### Subjects

**FIGURE 1:**
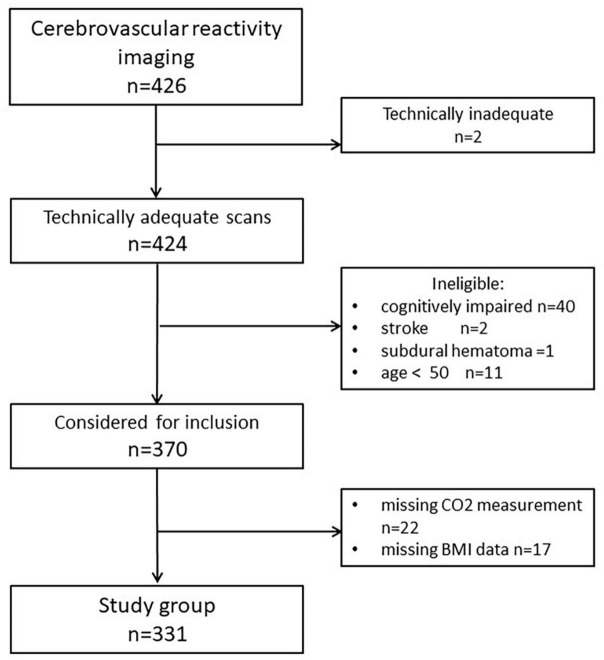
A flowchart describing the final study sample.

The study was a retrospective analysis of prospectively collected data. Subjects described in this report were enrolled for studies assessing the role of hippocampal hemodynamics as an early biomarker for ADs and cognitive decline among cognitively healthy elderly. They were prospectively recruited between January 2008 and November 2017 at the NYU Grossman School of Medicine, the former Center for Brain Health. Those who responded to the advertisement were either themselves interested in research participation and/or were the family members of cognitively impaired patients. All individuals signed Institutional Review Board-approved consent forms. Figure 1 presents exclusion criteria and participant flow: we reported on the final sample of 331 cognitively healthy subjects with CVR_CO2_ imaging. Participants diagnosed with probable dementia, mild cognitive impairment, and subjects with stroke and active substance abuse were excluded. Clinical diagnosis was based on a physician-administered interview, including ratings on the Brief Cognitive Rating Scale, the Global Deterioration Scale (Reisberg et al., 1993), and the Clinical Dementia Rating (Morris, 1993). Different data from this group were described previously (Glodzik et al., 2019).

### Clinical assessment

All subjects underwent medical, psychiatric, and neurological assessments, blood tests, ECG, and MRI examinations. Blood samples taken in a fasting state were examined for complete blood count, liver function tests, and metabolic and lipid panels. Since the studies contributing participant data to the center did not have perfectly uniform lab acquisition, only a subgroup of subjects had their plasma insulin (*n* = 280) assessed.

*Body mass index (BMI)* was calculated as weight/height^2^ [kilograms]/[meters]^2^. Obesity was defined based on Center for Disease Control and Prevention definition as BMI ≥ 30. Obesity or lack of thereof was not a criterion for recruitment into studies conducted at our center. *Diabetes mellitus* was established based on medical history, usage of glucose-lowering medication, or fasting glucose plasma level ≥ 126 mg/dl (American Diabetes Association., 2020).

*QUICKI* (Quantitative insulin sensitivity check index) (Katz et al., 2000) was calculated as:

*1/(Log10 (fasting insulin)* + *Log10 (fasting glucose)).*

*Insulin resistance (IR*) was defined as QUICKI ≤ 0.35.

Blood pressure was taken in a sitting position, after 5 min of rest (Muntner et al., 1979). It was measured on the left upper arm using a sphygmomanometer. *Medication:* The use of antihypertensive medications and statin drugs was recorded and used as yes/no categorical variables. *Hypertension (HTN)* was defined as current antihypertensive treatment or BP ≥ 140/90 mmHg (Chobonian et al., 2003).

### Magnetic resonance imaging

All magnetic resonance (MR) imaging scans were performed on the state-of-the-art 3T Prisma system (Siemens, Erlangen, Germany) equipped with a fast gradient system (rise time 200 T/m/s, peak strength 80 mT/m), a parallel transmit array, a zoomed image field-of-view selection, and enhanced parallel-receive array hardware. The imaging protocol consisted of sagittal T1-weighted Magnetization Prepared Rapid Acquisition Gradient Echo (MP-RAGE) [repetition time (TR) = 2,250 ms, echo time (TE) = 2.7 ms, inversion time (TI) = 900 ms, flip angle (FA) = 8°, slice thickness: 1.0 mm, field of view (FOV) = 200 mm],

Fluid Attenuation Inversion Recovery (FLAIR) (TR/TE/TI 9000/99/2,500 ms; FA 130°, slice thickness: 3.3 mm, FOV 220 mm), and locally developed high-resolution perfusion arterial spin labeling (ASL) sequence based on True-fisp balanced steady-state free precession (bSSFP) readout (TI = 1.2 s, FA = 50°, bandwidth = 977 Hz/pixel, 30 × 19.7 cm field of view, slice thickness of 6 mm and in-plane resolution 1.2 × 1.2 mm); (Glodzik et al., 2019).

It takes about 2.5 s to collect a tag/untag pair. We acquired one series of 24 tag/untag repetitions (48 images) during the baseline condition of 1 min. After initiating the rebreathing challenge that was continuously monitored with the capnograph, the second series (also 1 min) of alternating tag/untag images was acquired. The technical details are described in Supplementary material.

### MR image processing

#### Structural MRI processing

Gray matter (GM) and intracranial volumes (ICVs) were estimated using Statistical Parametric Mapping (SPM, version 8) with “New-Segment” extension (Ashburner and Friston, 2000). To match hemodynamic data, structural analyses were also focused on the hippocampus. Left and right hippocampal volumes were obtained with FreeSurfer version 6.0. (Fischl et al., 2002); they were presented as ratios to the ICV.

#### Cerebral blood flow sampling

**FIGURE 2:**
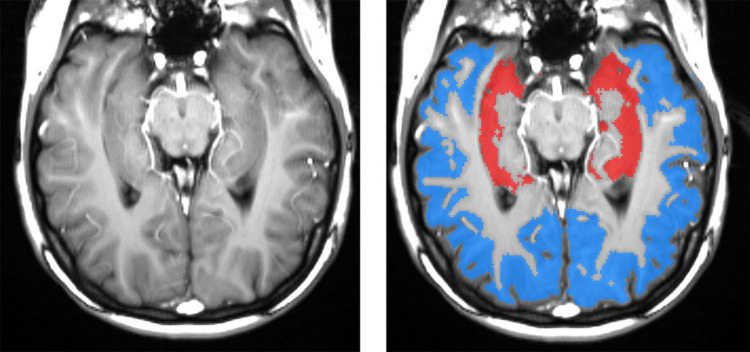
Left panel: spatial resolution and tissue contrast on a tagged (labeled) oblique balanced steady-state free precession image through the main body of the hippocampus. The right panel shows segmented masks for hippocampal (red) and cortical (blue) gray matters. High-resolution ASL enables an accurate definition of hippocampal gray matter, which is important because gray matter CBF is two to three times greater than that in the white matter. Including a variable fraction of white matter would contaminate results with significant errors. The cortical ROI encompasses mostly the temporal and parietal and, in some cases, also occipital cortices on the same axial slice that included both hippocampi.

To optimize hippocampal sampling, ASL was acquired using an oblique slice passing through the left and right hippocampi and the middle temporal gyrus (Glodzik et al., 2019). The hippocampal, cortical, and white matter (WM) regions of interest (ROIs) could be segmented with high accuracy directly on high-resolution ASL images (Figure 2). The WM region was used to calibrate slice profile error arising from the different inversion profiles of water in static tissues by the global and slice-selective inversion pulses. Thus, no CBF for WM was calculated. CBF was calculated in the left and right hippocampi and the cortex. Right and left hippocampal CBF data were averaged to create a mean referred to as “hippocampal CBF.” The cortical ROI encompassed the temporal, parietal, and, in some cases, also occipital cortices on the same axial slice that included both hippocampi. Additional information about ASL processing and sampling is presented in Supplementary material.

#### Cerebrovascular reactivity imaging

To estimate CVR, the CO_2_ level was increased using a rebreathing protocol (Rusinek et al., 2011). Subjects were asked to breathe through a mouthpiece and a respiratory tube. The rebreathing apparatus included a standard gas-anesthesia tube of 35 mm diameter and a custom-adjusted length. The subject’s nose was clamped to force inspiration of partially exhaled air. Oxygen saturation, heart rate, respiratory rate, and CO_2_ content in the expired air were monitored during image acquisition using a Medrad Veris system. Two consecutive acquisitions were performed at *Baseline*, where the subject breathed using only a mouthpiece, and at *Challenge*, where the participant had to breathe through a mouthpiece and a respiratory tube. This procedure is known to elicit mild hypercapnia (Glodzik et al., 2011). Indeed, the rebreathing challenge corresponded to a reliable end-tidal CO_2_ increase of 6 mmHg (refer to the “Results” section). We stopped breathing through a tube if oxygen saturation fell below 92% for more than 15 s or CO_2_ increased by more than 15 mmHg. The termination point was not standardized. Participants were not asked to hyperventilate. A subset of 129 subjects repeated the rebreathing examination through a tube on a different occasion. There was no difference in average CO_2_ change during the first and second rebreathing events. The ICC was moderate at a value equal to 0.5.

The CVR_CO2_ response to an increase in blood CO_2_ was calculated as a slope of the presumed linear relationship between CBF and end-tidal CO_2_:

*CVR*_CO2_ = ((CBF_challange_ – CBF_baseline_)/CBF_baseline_)*100)/**Δ** CO_2_.

where *CBF*_*challange*_ indicates CBF calculated during the session when subjects breathed through a respiratory tube and *CBF*_*baseline*_ indicates CBF calculated during the imaging session without the tube. The units of CBF were ml/100 g/min. **Δ** CO_2_ indicates the difference in end-tidal CO_2_ between two conditions. For hippocampi, baseline and challenge CBF represent an average over left and right.

### Statistical analyses

Categorical variables were compared with χ^2^-tests. *T*-test, paired *t*-test, or analysis of covariance (ANCOVA) was used to compare group means for continuous variables. When appropriate, we used non-parametric Mann-Whitney *U*-test or ranked ANCOVA. Correlations were evaluated using Pearson or Spearmen coefficients.

MRI data were acquired over the span of 9 years using a 3T magnet system that underwent hardware and software upgrades. We detected the inter-epoch variability in CBF, CVR_CO2_, and volume measurements. To avoid this fixed bias, the perfusion values were z-scored, recentered, and rescaled as previously described (Glodzik et al., 2019). In a similar way, we z-scored both GM and hippocampal volumes and CVR_CO2_ values separately by epoch and re-centered and rescaled them.

Finally, the average increase in CBF in response to 1 mmHg increase in carbon dioxide approximates 4 (Grüne et al., 2015) to over 6% (Coverdale et al., 2014). Thus, we excluded all subjects with CVR_CO2_ exceeding 10% on both high and low tails, as these outlier values were deemed to result from measurement error. This left 298 subjects for the analysis of both hippocampi and 325 subjects for the analysis of the cortex. Included and excluded subjects did not differ in age, sex, hypertension, or BMI.

Since this cutoff value may be considered too arbitrary, we repeated our CVR_CO2_ analyses after excluding subjects with values exceeding ± 2SD outside the mean. The results are presented in Supplementary material.

To compare resting and challenge the levels of vital parameters (within-subject factor) between obese and non-obese subjects or between men and women (between-subjects factor), we used the general linear model (GLM) repeated measures analysis.

The relationships between CVR_CO2_, CBF, brain volumes, and BMI were first examined using a linear regression model, in which brain metric was a dependent variable and BMI was used as a sole predictor. Whenever BMI was found to be significant in a model, it was further adjusted for age, sex, hypertension, and *BMI-sex* interaction. If the BMI-sex interaction was not found to be significant at a 0.10 level of significance, it was removed in a finalized model. If it was significant, we carried out additional analyses stratified by sex. Normality assumptions were checked for each model, and when necessary, analyses were repeated on ranked-transformed variables.

We presented here the *F*-value for the regression models, as well as unstandardized (B) and standardized (β) coefficients for individual predictors, and the corresponding *p*-values. Analyses were conducted in the entire group and repeated for subgroups of subjects without diabetes and for those without diabetes or IR.

Statistical significance was defined as a *p*-value < 0.05. SPSS (version 25, SPSS, Inc., Chicago, IL, United States) software was used for all analyses. Due to a large number of tests conducted, we used the Benjamini-Hochberg procedure to control the false discovery rate, which was set at 15%. The *p*-value < 0.05 was considered significant (for details, refer to Supplementary Table 6).

## Results

Our group of 331 subjects consisted of 60.4% women (age 68.8 ± 7.5 years; education 16.8 ± 2.2 years) and 39.6% men (age 70.4 ± 6.4 years; education 16.9 ± 2.4 years). Approximately 22% of them (*n* = 73) were obese. Clinical, demographic, and imaging characteristics of the study group are provided in Table 1, stratified by obesity status, in Supplementary Table 1, stratified by sex, and in Supplementary Table 1A, stratified by sex and obesity status.

**TABLE 1:** Baseline characteristics of the study group (*n* = 331), by obese status.

Variable	Obese (*n* = 73)	Non-obese (*n* = 258)	*P*
Age (years)	70.3 ± 6.8	69.2 ± 7.2	0.31
Sex (*n*, % women)	42, 58%	158, 61%	0.57
Education (years)	16.2 ± 2.6	17.0 ± 2.2	**0.02**
BMI	34.4 ± 3.9	24.4 ± 2.8	NA
SBP (mmHg)*^a^*	131.8 ± 16.7	122.6 ± 15.7	**<0.001**
DBP (mmHg)*^a^*	78.1 ± 10.4	72.7 ± 10.1	**<0.001**
Glucose*^b^* (mg/dL)	89.4 ± 17.2	82.8 ± 16.1	**0.001**
QUICKI*^c^*	0.35 ± 0.04	0.38 ± 0.04	**<0.001**
Insulin resistance*^c^* (*n*, %)	35, 58%	37, 17%	**<0.001**
Diabetes mellitus (*n*, %)	6, 8%	11, 4%	0.18
Antihypertensive medication (*n*, %)	32, 44%	80, 31%	**0.04**
Statins (*n*, %)	27, 37%	83, 32%	0.44
Hippocampal volume*^e^* (% ICV)	0.261 ± 0.003	0.260 ± 0.002	0.76
Gray matter volume*^e^* (% ICV)	39.6 ± 0.41	41.3 ± 0.21	**<0.001**
Hippocampal CBF*^f^* (ml/100 g/min)	63.2 ± 1.03	64.1 ± 0.55	0.42
Cortical CBF*^g^* (ml/100 g/min)	58.5 ± 0.67	58.5 ± 0.35	0.99
Hippocampal CVR_CO2_ (%)*^h^*	0.70 ± 2.70	1.54 ± 2.33	**0.01**
Cortical CVR_CO2_ (%)*^i^*	0.81 ± 1.54	1.06 ± 1.42	0.18
CO_2_ difference (mmHg)	5.7 ± 4.0	6.2 ± 3.9	0.36
Sat_*O*2_ difference (%)*^j^*	−0.30 ± 1.7	−0.48 ± 1.5	0.39
Respiratory rate difference (breaths/min)*^k^*	−0.53 ± 3.0	−0.50 ± 2.6	0.95
Heart rate difference (beats/min)*^l^*	0.34 ± 4.9	0.35 ± 3.1	0.98

BMI, body mass index; SBP, systolic blood pressure; DBP, diastolic blood pressure; QUICKI, quantitative insulin sensitivity check index; ICV, intracranial volume; CBF, cerebral blood flow; CVR_CO2_, cerebrovascular reactivity to carbon dioxide; Sat_O2_, oxygen saturation. Data are presented as mean ± standard deviation, p-values were obtained using the Mann-Whitney U-test, unless otherwise indicated. For categorical variables, χ^2^ was used.^a^Data available for 328 subjects: 72 obese and 256 non-obese.^b^Data available for 325 subjects: 71 obese and 254 non-obese.^c^Data available for 280 subjects: 60 obese and 220 non-obese. Comparisons of brain volumes, CBF, and CVR_CO2_ were performed with ANCOVA with initial adjustments for age, sex, and hypertension. Covariates were retained in the model only if significant.^e^Values presented as mean ± SE, a p-value from ANCOVA after accounting for age and sex.^f^Presented as mean ± SE, a p-value from ANCOVA after accounting for sex.^g^Presented as mean ± SE, a p-value from ANCOVA after accounting for sex and hypertension.^h^Data available for 298 subjects: 66 subjects with obesity and 232 non-obese subjects.^i^Data available for 325 subjects: 73 subjects with obesity and 252 non-obese subjects.^j^Data available for 310 subjects: 70 subjects with obesity and 240 non-obese subjects.^k^Data available for 306 subjects: 69 subjects with obesity and 237 non-obese subjects.^l^Data available for 308 subjects: 68 subjects with obesity and 240 non-obese subjects. Differences in CO_2_, SatO_2_, respiratory rate, and heart rate are between baseline and challenge conditions (rebreathing). p-values come from GML repeated measures analyses, with obesity status as the between-subjects factor; baseline and challenge vital signs values as the within-subjects factor. Significant p values were bold.

A comparison of physiologic readings between baseline and rebreathing challenge for the entire group showed that CO_2_ in the end-tidal air increased by 6.08 ± (standard deviation) 3.9 mmHg, heart rate (HR) increased by 0.34 ± 3.5 beats per minute, respiratory rate (RR) decreased by 0.51 ± 2.7 breaths per minute, and oxygen saturation (Sat_*O*2_) decreased by 0.44 ± 1.5%. The paired *t*-test revealed that all differences, except HR, were significant. GLM repeated measures analysis with obesity status as a between-subjects factor showed that changes in the above physiological parameters in response to rebreathing did not differ between subjects who are obese and non-obese (Table 1).

Hippocampal CBF was higher than the cortical one (*t*-test *p* < 0.001). Similarly, the hippocampus had higher CVR_CO2_ than the cortex (*p* = 0.03).

In the following sections, we presented relationships between BMI and brain measures (volume, CBF, CVR) for the hippocampus and the cortex separately. Within each region, we performed analyses in the entire group and then in subgroups of participants without diabetes and those without both diabetes and insulin resistance.

### Hippocampus

**FIGURE 3:**
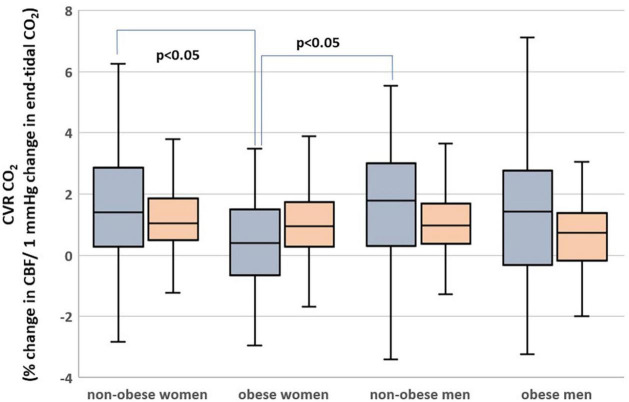
Hippocampal (gray) and cortical (orange) cerebrovascular reactivity to CO_2_ (CVR_*CO2*_) in men and women with obesity and non-obese men and women.

Subjects who are obese had significantly lower hippocampal CVR_CO2_ (Table 1). In univariate analyses, BMI was associated with CVR_CO2_ [model’s *F*_(1,297)_ = 4.5, *p* = 0.04] but not with hippocampal CBF or volume. In the fully adjusted model [*F*_(5,297)_ = 2.3, *p* = 0.04], *BMI-sex* interaction was significant (Table 2). Further analyses stratified by sex showed that BMI was significantly associated with hippocampal CVR_CO2_ only in women [model’s *F*_(3,184)_ = 4.89, *p* = 0.003]. The higher the BMI, the lower the CVR_CO2_ (Table 2 and Figure 3).

**TABLE 2:** Linear regression models predicting hippocampal CVR_CO2_ in the entire group and among women and men separately.

Variable	Unstandardized B	Standardized β	*P*-value	95% CI for B
**Entire group**				
BMI	−0.06	−0.12	**0.04**	−0.11, −0.004
Age	−0.03	−0.10	0.10	−0.07, 0.01
Sex	2.64	0.53	0.11	−0.60, 5.87
Hypertension	−0.28	−0.06	0.34	−0.86, 0.30
BMI	0.03	0.06	0.60	−0.08, 0.13
BMI*sex	−0.11	−0.58	0.08	−0.22, 0.01
**Women**				
Age	−0.04	−0.13	0.07	−0.08, 0.003
Hypertension	−0.35	−0.08	0.29	−1.003, 0.31
BMI	−0.08	−0.20	**0.008**	−0.13, −0.02
**Men**				
Age	−0.02	−0.05	0.65	−0.10, 0.07
Hypertension	−0.19	−0.03	0.74	−1.29, 0.92
BMI	0.02	0.04	0.70	−0.10, 0.15

Significant *p* values were bold.

### Relationship between body mass index and hippocampus in subjects without diabetes

In subjects without diabetes (*n* = 284), BMI was associated with hippocampal CVR_CO2_ [model’s *F*_(1; 283)_ = 4.0, *p* = 0.046] but not with hippocampal CBF or volume. In a fully adjusted analysis, *BMI-sex* interaction was statistically significant (Supplementary Table 2). Analyses stratified by sex again revealed the BMI-hippocampal CVR_CO2_ relationship only in women [model’s *F*_(3,181)_ = 4.85, *p* = 0.003] (Supplementary Table 2).

### Relationship between body mass index and hippocampus in subjects without diabetes and insulin resistance

Information about insulin resistance was available for 280 subjects. Two hundred eight did not show insulin resistance and 186 out of 208 had hippocampal CVR_CO2_ data. There was a negative relationship between BMI and hippocampal CVR_CO2_ [models’ *F*_(1,185)_ = 3.9, *p* = 0.049]. Further adjustments for age, sex, and hypertension did not show that these covariates were significant (Supplementary Table 3). The *BMI-sex* interaction did not contribute to the model. BMI was not associated with hippocampal CBF or volume.

Analyses using CVR_CO2_ values after taking outliers 2 standard deviations outside the mean (*n* = 315) are presented in Supplementary Table 7. They yielded similar results.

### Cortex

Body mass index was associated with GM volume [model’s *F*_(1,330)_ = 19.5, *p* < 0.001], but not with CVR_CO2_ or CBF. In the fully adjusted model [*F*_(4,330)_ = 35.9, *p* < 0.001], age and sex were also statistically significant (Table 3). The *BMI-sex* interaction did not contribute to the model. Higher BMI and older age were associated with lower GM volume. Women had higher GM volumes than men.

**TABLE 3:** Linear regression models predicting GM volumes.

Variable	Unstandardized B	Standardized β	*P*-value	95% CI for B
BMI	−0.19	−0.24	**<0.001**	−0.27, −0.10
Age	−0.27	−0.46	**<0.001**	−0.32, −0.21
Sex	1.12	0.14	**0.004**	0.36, 1.87
Hypertension	−0.16	−0.02	0.69	−0.94, 0.62
BMI	−0.15	−0.20	**<0.001**	−0.23, −0.08

Significant *p* values were bold.

### Relationship between body mass index and cortex in subjects without diabetes

In subjects without diabetes (*n* = 314), univariate analyses showed that BMI was inversely associated with GM volume [models’ *F*_(1,313)_ = 21.0, *p* < 0.001] but not with cortical CVR_CO2_ or CBF. In the fully adjusted model [*F*_(4,313)_ = 36.6, *p* < 0.001], age and sex were statistically significant (Supplementary Table 4). Higher BMI and older age were associated with higher atrophy. Women had higher GM volumes than men.

### Relationship between body mass index and cortex in subjects without diabetes and insulin resistance

In this subgroup (*n* = 208), BMI was inversely associated with GM volume [models’ *F*_(1,207_ = 5.1, *p* = 0.03] in univariate analysis. However, this relationship was no longer significant in the fully adjusted model [*F*_(4,207)_ = 21.7, *p* < 0.001] (Supplementary Table 5). BMI was not associated with cortical CVR_CO2_ or CBF.

Analyses using CVR_CO2_ values after taking outliers 2 standard deviations outside the mean (*n* = 313) yielded similar results: no relationship was found between BMI and cortical CVR_CO2_.

## Discussion

In a group of cognitively unimpaired middle-aged and older adults, higher BMI was associated with lower hippocampal CVR_CO2_. This relationship was present in women. Women are at a greater risk of developing dementia than men (Seshadri et al., 1997). In addition, and most relevant to our study, obesity seems to play a stronger role as a dementia risk factor in women (Whitmer et al., 2005; Ma et al., 2020). Women differ from men in terms of body fat distribution, metabolic and hormonal regulation of fat deposition, and adipocytes function (Pradhan, 2014). There is also evidence that the hemodynamic response to CO_2_ challenge differs between sexes, although the direction of these differences is not settled and may depend on age (Deegan et al., 2011; Urback et al., 2018). Finally, our previous study showed that higher BMI was related to amyloid deposition in women but not in men (Glodzik et al., 2016). All these prompted us to stratify analyses based on sex. In our group, women had a somewhat better profile of risk factors than men, especially less insulin resistance and less diabetes, even within the obese subgroup (although the differences between obese women and obese men are not significant). Women had higher brain volumes and higher CBF; however, women who were obese had the lowest hippocampal CVR_CO2_, and BMI-hippocampal CVR_CO2_ relationship was evident among women. Our finding, although novel in the brain, is not without precedent in the periphery. In an earlier study, measures of fat distribution were inversely associated with endothelium-dependent and flow-mediated vasodilation, and these relationships were stronger in women than in men (Lind et al., 2019). There is also evidence indicating that women are particularly prone to developing obesity-related endothelial impairment (Suboc et al., 2013). Although mechanisms of this vulnerability are not fully explained, some suggested that higher expression and activation of endothelial mineralocorticoid receptors in women may be a culprit (Faulkner et al., 1979). An alternative explanation could relate to sex hormone levels, which we did not measure. Obesity is related to an increase in androgen level in women but not in men. Since androgens are known vasoconstrictors, we speculate that increased androgens might have contributed to the observed association (Abi-Ghanem et al., 2020). It is also possible that, since in our group there were fewer men than women, there was not enough power to detect the relationship. Endothelial injury is a plausible cause of impaired vasoreactivity (Lavi et al., 2006). High BMI may affect endothelium in a few ways. Endothelial progenitor cells of individuals with obesity release less proangiogenic factors and are more susceptible to apoptotic stimuli (MacEneaney et al., 2010). Second, high BMI results in a persistent proinflammatory state, leading in turn to endothelial damage (Rocha and Libby, 2009).

Earlier research indicates that both diabetes (Last et al., 2007) and insulin resistance, (Frosch et al., 2017) linked to higher BMI, are characterized by gray matter CVR_CO2_ impairment. Others reported a negative association between global measures of vasoreactivity, namely, the breath holding index and BMI (Rodríguez-Flores et al., 2014). Our novel observation that a negative BMI-CVR_CO2_ relationship is present in the hippocampus supports previous epidemiological evidence that obesity is a risk factor for dementia (Kivipelto et al., 2005) and neurodegeneration (Gottesman et al., 2017). An earlier study suggested that it was not obesity itself, but rather its metabolic complication, that is, insulin resistance that was responsible for CVR_CO2_ impairment (Frosch et al., 2017). Our study, conducted in a larger and older group, demonstrates a significant inverse relationship between BMI and hippocampal CVR_CO2_ even after excluding subjects with diabetes and insulin resistance.

In contrast to earlier findings of reduced CBF with increased body weight (Amen et al., 2020), we found no relationship between BMI and hippocampal blood flow in our group. Hippocampal volume and BMI were also not related. This is somewhat unexpected but may be consistent with a previous study, which found larger hippocampi in elderly subjects with obesity (Widya et al., 2011). On the other hand, several other reports linked obesity with hippocampal atrophy (Cherbuin et al., 2015; Lynch et al., 2021). It is tempting to conclude that functional impairment, detectable in the condition of increased demand, preceded deficit in resting CBF and structural damage. However, our observation that BMI was associated with GM volume, but not cortical CBF or CVR_CO2_, does not fully support this hypothesis. Our method of assessing cortical perfusion only partially (6 mm slab at the level of the middle temporal lobe) could be responsible for these discrepancies. While hippocampal regions were matched for volume, flow and vasoreactivity, we measured global GM volume from the 3D T1W sequence, but CBF and CVR_CO2_ were derived from a single slice used in the ASL protocol.

We found that hippocampal perfusion was higher than cortical CBF. The same was true for CVR_CO2_. Possibly, higher vascular density in the hippocampus as compared to the cortex (Rane et al., 2016) is responsible for this finding. The spillover of ASL signals from big vessels passing near the hippocampus (larger than penetrating arteries in the temporal cortex) could also be the reason for this discrepancy. Finally, it is tempting to speculate that heightened vascular demand puts the hippocampus at a greater risk in the state of challenge.

Overall, we offer that BMI-CVR_CO2_ relationship in the hippocampus, but not in the surrounding cortex, provides further evidence for hippocampal sensitivity to inflammatory and ischemic damage (Bartsch and Wulff, 2015). In the end, the ultimate proof of the causal relationship between BMI and CVR would be a prospective intervention aimed at BMI reduction.

There are a few limitations to our study. First, and most importantly, it was previously shown that computer-controlled, prospective, end-tidal targeting is an optimal and reliable method of controlling the level of exhaled CO_2_ (Fisher, 2016). Our rebreathing method produces a rather mild hypercapnia, the termination point not was standardized, and participants were not asked to hyperventilate. All these factors might have resulted in a weak stimulus in our measurements.

The hippocampus is a small structure, and thus, its measurements are intrinsically more susceptible to noise and error, as evidenced by higher numbers of subjects excluded from the analysis of this region. Second, our group was unbalanced: it contained more women, who were younger and healthier. This, however, should have worked against our hypothesis, preventing us from showing BMI-vasoreactivity association among women. We did not assess the level of physical activity in our participants. Similarly, we do not have more precise measures of body composition. Possibly assessing relationships between brain metrics and fat mass or fat-free mass could have revealed more informative associations.

Our subjects were cognitively healthy, middle-aged, or older; thus, we do not know whether we can generalize findings to cognitively impaired individuals in different age groups. Finally, cross-sectional associations cannot be substituted for causal relationships. We do not know how many subjects developed dementia and whether these subjects with reduced hippocampal CVR_CO2_ were at a higher risk.

Despite these shortcomings, we observed that among cognitively healthy middle-age and older adults, higher BMI was associated with lower hippocampal CVR_CO2_. This phenomenon persisted after excluding subjects with insulin resistance and was seen predominantly in women. Altogether, these findings lend support to the notion that obesity is a risk factor for hippocampal hemodynamic impairment and suggest targeting obesity as an important prevention strategy. Prospective studies assessing the effects of weight loss on brain hemodynamic measures are warranted.

## Data availability statement

The data that support the findings of this study are available from the corresponding author on reasonable request.

## Ethics statement

The studies involving human participants were reviewed and approved by the Institutional Review Board at NYU Grossman School of Medicine. The patients/participants provided their written informed consent to participate.

## Author contributions

LG, HR, TB, PS, and ML: drafting and revision of manuscript for content (incl. medical writing), study concept and design, data analysis and interpretation, and major role in data acquisition. YL: drafting and revision of manuscript for content (incl. medical writing), study concept and design, and data analysis and interpretation. ES: drafting and revision of manuscript for content, and data analysis and interpretation. RO: drafting and revision of manuscript for content (incl. medical writing), and data analysis and interpretation. AB, CW, and TM: drafting and revision of manuscript for content (incl. medical writing). ET and PH: drafting and revision of manuscript for content, and major role in data acquisition. All authors contributed to the article and approved the submitted version.
