# Supplementary material for: Higher body mass index is associated with worse hippocampal vasoreactivity to carbon dioxide
Source: Front Aging Neurosci. 2022 Sep 7;14:948470. doi: 10.3389/fnagi.2022.948470 (PMC9491849; doi:10.3389/fnagi.2022.948470)
Supplement: Supplementary file 1 [file Data_Sheet_1.docx]

*Imaging*

*Arterial spin labeling (ASL) acquisition.* We used a pulsed ASL sequence combining a flow-sensitive alternating inversion-recovery (FAIR) labeling scheme with balanced steady-state free precession (bSSFP) readout ^1,2^ to achieve better spatial resolution and lower susceptibility artifacts. Data were acquired in a single shot after the inversion pulse. The TR between successive inversion pulses was 3s. To improve the signal to noise ratio, 24 repetitions were performed, alternating between slice-selective and non-selective inversions. The first 4 repetitions were excluded from the analysis, to ensure that the system had reached a quasi-steady state.

It takes about about 2.5 seconds to collect a tag/untag pair. We collected one image during baseline condition: 24 repetitions tag/untag - 1 minute, and second image during challenge condition (also 1 minute).

*ASL processing.* Perfusion was estimated in hippocampal and cortical regions of interest (ROIs). Cerebral flood flow (CBF) was computed using a “standard model” approximation: ^3^

$CBF=\frac{\Delta M\lambda e^{TI/T_{1}}}{2\sigma TIM_{0}}=g\Delta M$ [1]

where Δ*M* is the difference between tagged and untagged signal in the given ROI, λ is the blood-tissue water partition coefficient (assumed 0.9 ml/g), σ is the inversion efficiency, *TI* is the inversion delay (1200 ms), and *M*_0_ is the equilibrium magnetization. Because at 1.2 seconds after labeling most of the magnetization remains in the blood, ^4^ we have used the relaxation time of the arterial blood (*T*_1_=1930 ms at 3T) ^5^ in the above equation.

The FAIR ASL method avoids uncertainties of a variable blood transit, due to the placement of the labeling slab around each slice. However, it also assumes that the global inversion pulse inverts water in static tissue to the same extent as the slice-selective inversion pulse, which is not accurate due to imperfect slice profiles and B0 field inhomogeneity. To compensate for this limitation, we used the signal in normally appearing white matter (WM) to calibrate slice profile error. ^2,6^

*CBF sampling.* Hippocampal, cortical, and white mater (WM) region of interest (ROIs) were defined directly on high-resolution ASL images (to minimize partial volume errors), using an in-house-developed software (<https://firevoxel.org>) ^7^ Cortical ROI encompassed temporal, parietal and in some cases also occipital cortex (Figure 1). The process entailed: a) choosing a seed region in GM and WM, b) constructing a WM ROI within 10% of the WM seed, restricted to the largest connected components and refined by automatic boundary erosion, c) constructing GM ROI by intensity thresholding followed by automatic boundary erosion and removal of nonbrain tissue, ^2^ d) delineating right and left hippocampal regions. Each ROI was visually confirmed by investigators knowledgeable about the studied anatomy. A batch process was then run to generate GM and hippocampal CBF. In the final step, all voxels with CBF >150 ml/(100g min) were deemed to contain large blood vessels and were excluded from ROIs. ^6^

**Supplemental Table 1.** Baseline characteristics of the study group (n=331), by sex.

| **Variable** | **Women (n=200)** | **Men**  **(n=131)** | **p** |
| --- | --- | --- | --- |
| **Age (years)** | 68.8 ± 7.5 | 70.4 ± 6.4 | **0.046** |
| **Education (years)** | 16.8 ± 2.2 | 16.9 ± 2.4 | 0.77 |
| **BMI** | 26.2 ± 5.6 | 27.2 ± 4.4 | **0.004** |
| **Obese (n, %)** | 42, 21% | 31, 24% | 0.57 |
| **SBP (mmHg)^a^** | 122.8± 16.9 | 127.3 ± 15.0 | **0.013** |
| **DBP (mmHg)^a^** | 73.8 ± 10.3 | 73.9 ± 10.5 | 0.87 |
| **Glucose^b^ (mg/dL)** | 82.1 ± 16.2 | 87.5 ± 16.7 | **0.002** |
| **QUICKI**^c^ | 0.38 ± 0.04 | 0.37 ± 0.04 | **0.006** |
| **Insulin resistance**^c^ **(n, %)** | 40, 23% | 32, 30% | 0.20 |
| **Diabetes mellitus (n, %)** | 4, 2% | 13, 10% | **0.001** |
| **Antihypertensive medication (n, %)** | 58, 29% | 54, 41% | **0.02** |
| **Statins (n, %)** | 57, 29% | 53, 41% | **0.02** |
|  |  |  |  |
| **Hippocampal volume**^e^ **(%ICV)** | 0.265 ± 0.002 | 0.253 ± 0.002 | **<0.001** |
| **Gray matter volume**^f^ **(% ICV)** | 41.4 ± 0.24 | 40.3 ± 0.30 | **0.003** |
| **Hippocampal CBF**^g^ **(ml/100g/min)** | 65.4 ± 9.4 | 61.8 ± 7.8 | **<0.001** |
| **Cortical CBF**^h^ **(ml/100g/min)** | 59.7 ± 0.41 | 56.6 ± 0.50 | **<0.001** |
| **Hippocampal CVR_CO2_ (%)**^i^ | 1.30 ± 0.18 | 1.45 ± 0.23 | 0.60 |
| **Cortical CVR_CO2_ (%)**^j^ | 1.10 ± 1.50 | 0.86 ± 1.40 | 0.17 |
|  |  |  |  |
| **CO_2_ difference (mmHg)** | 6.4 ± 3.8 | 5.7 ± 4.2 | 0.12 |
| **Sat_O2_ difference (%)**^k^ | -0.40 ± 1.7 | -0.51 ± 1.2 | 0.55 |
| **Respiratory rate difference (breaths/min)**^l^ | -0.55 ± 2.7 | -0.45 ± 2.7 | 0.76 |
| **Heart rate difference (beats/min)**^m^ | 0.54 ± 4.1 | 0.04 ± 2.5 | 0.23 |

BMI: Body mass index, SBP: systolic blood pressure, DBP: diastolic blood pressure, QUICKI: Quantitative insulin sensitivity check index, ICV: intracranial volume, CBF: cerebral blood flow, CVR_CO2_: cerebrovascular reactivity to carbon dioxide, Sat_O2_: oxygen saturation

Data is presented as mean ± standard deviation, p values come U Mann-Whitney test, unless otherwise indicated. For categorical variables χ^2^ was used.

^a^ data available for 328 subjects: 197 women and 131 men

^b^ data available for 325 subjects: 195 women and 130 men

^c^ data available for 280 subjects: 174 women and 106 men

^d^ data available for 125 subjects: 44 women and 81 men

Comparisons of brain volumes, CBF and CVR_CO2_ were done with ANCOVA with initial adjustments for age, BMI and hypertension. Covariates were retained in the model only if significant. If no covariates were significant and residuals were not normally distributed U Mann-Whitney test was used.

^e^ values presented as mean ± SE, p value from ANCOVA after accounting for age

^f^ values presented as mean ± SE, p value from ANCOVA after accounting for age and BMI

^g^ p value from t-test

^h^ values presented as mean ± SE, p value from ANCOVA after accounting for hypertension

^i^ data available for 298 subjects: 185 women and 113 men, values presented as mean ± SE, p value from ANCOVA after accounting for age and BMI

^j^ data available for 325 subjects: 198 women and 127 men

Differences in CO_2_, SatO_2_, respiratory rate and heart rate are between baseline and challenge condition (re-breathing). P-values come from GML repeated measures analyses, with sex as between subjects factor; baseline and challenge vital signs values as within subject factor.

^k^ data available for 310 subjects: 188 women and 122 men

^l^ data available for 306 subjects: 187 women and 119 men

^m^ data available for 308 subjects: 187 women and 121 men

**Supplemental Table 1A.** Baseline characteristics of the study group (n=331), by sex and obese status.

| **Variable** | **Obese Women (n=42)** | **Obese**  **Men**  **(n=31)** | **Non-obese**  **Women**  **(n=158)** | **Non-obese Men**  **(n=100)** | **p** |
| --- | --- | --- | --- | --- | --- |
| **Age (years)** | 70.2 ± 6.5 | 70.4 ± 7.1 | 68.4 ± 7.7 | 70.4 ± 6.1 | 0.12 |
| **Education (years)** | 16.2 ± 2.6 | 16.2 ± 2.7 | 17.0 ± 2.1 | 17.1 ± 2.3 | 0.13 |
| **BMI** | 35.2±4.2*† | 33.4±3.3*† | 23.8±2.9* | 25.3±2.5 | **<0.001** |
| **SBP (mmHg)^a^** | 131.9±17.1† | 131.6±16.4† | 120.4±16.1* | 125.9±14.4 | **<0.001** |
| **DBP (mmHg)^a^** | 77.3±10.4* | 79.1±10.6† | 72.9±10.1 | 72.4±10.1 | **0.002** |
| **Glucose^b^ (mg/dL)** | 86.9±15.4 | 92.8±19.1† | 80.8±16.2* | 85.9±15.6 | **<0.001** |
| **QUICKI**^c^ | 0.36±0.04*† | 0.34±0.03*† | 0.39±0.04 | 0.38±0.04 | **<0.001** |
| **Insulin resistance**^c^ **(n, %)** | 18, 51% | 17, 68% | 22, 16% | 15, 19% | **<0.001** |
| **Diabetes mellitus (n, %)** | 1, 2% | 5, 16% | 3, 2% | 8, 8% | **0.006** |
| **Antihypertensive medication (n, %)** | 14, 33% | 18, 58% | 44, 29% | 36, 36% | **0.01** |
| **Statins (n, %)** | 12, 29% | 15, 48% | 45, 28% | 38, 38% | 0.10 |
|  |  |  |  |  |  |
| **Hippocampal volume**^e^ **(%ICV)** | 0.265±0.004 | 0.255±0.004 | 0.265±0.002* | 0.253±0.004 | **<0.001** |
| **Gray matter volume**^e^  **(% ICV)** | 40.1 ± 0.53† | 40.0 ± 0.62† | 41.8 ± 0.27* | 40.5 ± 0.34 | **<0.001** |
| **Hippocampal CBF**^e^ **(ml/100g/min)** | 64.5 ± 8.8 | 61.3 ± 8.2 | 65.6 ± 9.5* | 61.9 ± 7.2 | **0.003** |
| **Cortical CBF**^f^ **(ml/100g/min)** | 59.1 ± 0.87* | 57.4 ± 1.03 | 59.8 ± 0.45* | 56.4 ± 0.57 | **<0.001** |
| **Hippocampal CVR_CO2_ (%)**^g^ | 0.46±2.10†* | 1.07±3.45 | 1.58±2.16 | 1.48±2.61 | **0.01** |
| **Cortical CVR_CO2_ (%)**^h^ | 0.93 ± 1.58 | 0.66 ± 1.50 | 1.15±1.46 | 0.93±1.34 | 0.31 |
|  |  |  |  |  |  |
| **CO_2_ difference (mmHg)** | 6.6 ± 3.4 | 4.5 ± 4.3 | 6.3 ± 3.8 | 6.0 ± 4.1 | 0.10 |
| **Sat_O2_ difference (%)**^i^ | -0.21 ± 1.9 | -0.41 ± 1.3 | -0.45 ± 1.7 | -0.54 ± 1.2 | 0.75 |
| **Respiratory rate difference (breaths/min)**^i^ | -0.53 ± 2.9 | -0.52 ± 3.2 | -0.55 ± 2.7 | -0.42 ± 2.5 | 0.98 |
| **Heart rate difference (beats/min)**^j^ | 0.23 ± 6.3 | 0.49 ± 2.2 | 0.62 ± 3.3 | -0.10 ± 2.5 | 0.23 |

BMI: Body mass index, SBP: systolic blood pressure, DBP: diastolic blood pressure, QUICKI: Quantitative insulin sensitivity check index, ICV: intracranial volume, CBF: cerebral blood flow, CVR_CO2_: cerebrovascular reactivity to carbon dioxide, Sat_O2_: oxygen saturation

Data is presented as mean ± standard deviation, p values come Kruskal-Wallis ANOVA, unless otherwise indicated. For categorical variables χ^2^ was used.

^a^ data available for 328 subjects: 41 obese women, 31 obese men, 156 non-obese women, 100 non-obese men

^b^ data available for 325 subjects: 41 obese women, 30 obese men, 154 non-obese women, 100 non-obese men

^c^ data available for 280 subjects: 35 obese women, 25 obese men, 139 non-obese women, 81 non-obese men

^d^ data available for 125 subjects: 16 obese women, 9 obese men, 65 non-obese women, 35 non-obese men

Brain metrics were initially compared using ANCOVA with age and hypertension as covariates in initial models. The most parsimonious models were chosen. If no covariates were significant and residuals were not normally distributed Kruskal-Wallis ANOVA was used.

^e^ p value from ANOVA

^f^ values presented as mean ± SE, p value from ANCOVA after accounting for age

^g^ data available for 298 subjects: 40 obese women, 26 obese men, 145 non-obese women, 87 non-obese men

^h^ data available for 325 subjects: 42 obese women, 31 obese men, 156 non-obese women, 96 non-obese men

Differences in CO_2_, SatO_2_, respiratory rate and heart rate are between baseline and challenge condition (re-breathing). P-values come from GML repeated measures analyses, with group (by sex and obesity status) as between subjects factor; baseline and challenge vital signs values as within subject factor.

^i^ data available for 310 subjects: 39 obese women, 31 obese men, 149 non-obese women, 91 non-obese men

^j^ data available for 306 subjects: 39 obese women, 30 obese men, 148 non-obese women, 89 non-obese men

^k^ data available for 308 subjects: 38 obese women, 30 obese men, 149 non-obese women, 91 non-obese men

For comparison between groups:

*Different from non-obese men at <0.05 corrected (Bonferroni)

† Different from non-obese women at <0.05 corrected

**Supplemental Table 2.** Linear regression models predicting hippocampal CVR_CO2_  in the subgroup without diabetes (n=284), and among women and men separately.

| **Variable** | **Unstandardized B** | **Standardized β** | **p-value** | **95% CI for B** |
| --- | --- | --- | --- | --- |
| Entire group | | | | |
| BMI | -0.06 | -0.12 | **0.046** | -0.11, -0.001 |
|  | | | | |
| age | -0.03 | -0.10 | 0.10 | -0.08, 0.01 |
| sex | 3.10 | 0.61 | 0.07 | -0.23, 6.45 |
| hypertension | -0.36 | -0.07 | 0.24 | -0.96, 0.24 |
| BMI | 0.04 | 0.09 | 0.42 | -0.06, 0.15 |
| *BMI*sex* | -0.12 | -0.64 | 0.06 | -0.24, 0.004 |
| Women | | | | |
| age | -0.04 | -0.14 | 0.06 | -0.08, 0.002 |
| hypertension | -0.34 | -0.08 | 0.32 | -1.01, 0.33 |
| BMI | -0.08 | -0.20 | **0.01** | -0.13, -0.02 |
| Men | | | | |
| age | -0.02 | -0.04 | 0.71 | -0.11, 0.08 |
| hypertension | -0.40 | -0.07 | 0.51 | -1.58, 0.80 |
| BMI | 0.04 | 0.07 | 0.51 | -0.09, 0.17 |

**Supplemental Table 3.** Linear regression models predicting hippocampal CVR_CO2_  in the subgroup without diabetes and insulin resistance (n=186).

| **Variable** | **Unstandardized B** | **Standardized β** | **p-value** | **95% CI for B** |
| --- | --- | --- | --- | --- |
| BMI | -0.08 | -0.15 | **0.049** | -0.16, -0.000 |
|  | | | | |
| age | -0.04 | -0.11 | 0.15 | -0.08, 0.01 |
| sex | -0.03 | -0.01 | 0.93 | -0.77, 0.71 |
| hypertension | 0.14 | 0.03 | 0.71 | -0.60, 0.87 |
| BMI | -0.07 | -0.14 | 0.073 | -0.15, 0.006 |

**Supplemental Table 4.** Linear regression models predicting GM volume in the subgroup without diabetes (n=314).

| **Variable** | **Unstandardized B** | **Standardized β** | **p-value** | **95% CI for B** |
| --- | --- | --- | --- | --- |
| BMI | -0.20 | -0.25 | **<0.001** | -0.28, -0.11 |
|  | | | | |
| age | -0.28 | -0.49 | **<0.001** | -0.33, -0.23 |
| sex | 0.95 | 0.11 | **0.02** | 0.16, 1.73 |
| hypertension | 0.007 | 0.001 | 0.98 | -0.79, 0.80 |
| BMI | -0.15 | -0.22 | **<0.001** | -0.24, -0.10 |

**Supplemental Table 5.** Linear regression models predicting GM volume in the subgroup group without diabetes and insulin resistance (n=208).

| **Variable** | **Unstandardized B** | **Standardized β** | **p-value** | **95% CI for B** |
| --- | --- | --- | --- | --- |
| BMI | -0.12 | -0.16 | **0.025** | -0.23, -0.02 |
|  | | | | |
| age | -0.25 | -0.49 | **<0.001** | -0.30, -0.19 |
| sex | 0.96 | 0.13 | **0.04** | 0.63, 1.86 |
| hypertension | -0.33 | -0.04 | 0.48 | -1.24, 0.58 |
| BMI | -0.07 | -0.09 | 0.15 | -0.16, 0.03 |

**Supplemental Table 6.** Benjamini-Hochberg procedure. Tests performed to assess relationships between BMI and hippocampal and cortical metrics. The p-value ≤0.049 was considered significant.

| **Test** | **p-value** | **Rank** | **Critical value at Q=15%** |
| --- | --- | --- | --- |
| BMI – GM volume  (subjects w/o DB2) | 0.000007 | 1 | 0.005357 |
| BMI – GM volume (subjects w/o DB2),  fully adjusted model | 0.00001 | 2 | 0.010714 |
| BMI – GM volume | 0.000014 | 3 | 0.016071 |
| BMI – GM volume, fully adjusted model | 0.000051 | 4 | 0.021429 |
| BMI – hippocampal CVR (women),  fully adjusted model | 0.008 | 5 | 0.026786 |
| BMI – hippocampal CVR (women w/o DB2), fully adjusted model | 0.01 | 6 | 0.032143 |
| BMI – GM volume (subjects w/o IR) | 0.025 | 7 | 0.0375 |
| BMI – hippocampal CVR | 0.036 | 8 | 0.042857 |
| BMI – hippocampal CVR  (subjects w/o DB2) | 0.046 | 9 | 0.048214 |
| BMI – hippocampal CVR  (subjects w/o IR) | 0.049 | 10 | 0.053571 |
| BMI – hippocampal CVR (subjects w/o IR),  fully adjusted model | 0.73 | 11 | 0.058929 |
| BMI – hippocampal volume  (subjects w/o DB2) | 0.076 | 12 | 0.064286 |
| BMI – GM CBF  (subjects w/o DB2) | 0.079 | 13 | 0.069643 |
| BMI – hippocampal volume | 0.096 | 14 | 0.075 |
| BMI – GM CBF | 0.097 | 15 | 0.080357 |
| BMI – GM volume (subjects w/o IR),  fully adjusted model | 0.148 | 16 | 0.085714 |
| BMI – hippocampal CBF | 0.167 | 17 | 0.091071 |
| BMI – hippocampal CBF  (subjects w/o DB2) | 0.165 | 18 | 0.096429 |
| BMI – hippocampal CBF  (subjects w/o IR) | 0.167 | 19 | 0.101786 |
| BMI – hippocampal volume  (subjects w/o IR) | 0.213 | 20 | 0.107143 |
| BMI – GM CVR  (subjects w/o DB2) | 0.34 | 21 | 0.1125 |
| BMI – GM CVR  (subjects w/o IR) | 0.37 | 22 | 0.117857 |
| BMI – GM CVR | 0.40 | 23 | 0.123214 |
| BMI – hippocampal CVR (subjects w/o DB2), fully adjusted model | 0.42 | 24 | 0.128571 |
| BMI – hippocampal CVR (men w/o DB2), fully adjusted model | 0.51 | 25 | 0.133929 |
| BMI – GM CBF  (subjects w/o IR) | 0.57 | 26 | 0.139286 |
| BMI – hippocampal CVR, fully adjusted model | 0.60 | 27 | 0.144643 |
| BMI – hippocampal CVR (women),  fully adjusted model | 0.70 | 28 | 0.15 |

Q: false discovery rate, GM: gray matter, DB2: diabetes mellitus type 2, CVR: cerebrovascular reactivity, IR: insulin resistance, CBF: cerebral blood flow, fully adjusted model includes sex, age, hypertension and interaction between sex and BMI, if interaction was significant at p<0.10. That was a case for 2 models: BMI – hippocampal CVR model in the entire group and in subjects without DB2.

***Hippocampal analyses using CVR_CO2_ values after taking outliers 2 standard deviations outside the mean (n=315)***

When we analyzed data using CVR_CO2_ values after taking outliers 2 standard deviations outside the mean, BMI was marginally associated with CVR_CO2_ in the univariate analysis (model’s F_1,314_=3.04, p=0.08). In the fully adjusted model (F_5,314_=2.9, p=0.01) *BMI-sex* interaction was significant (Table S7). Further analyses stratified by sex showed that BMI was significantly associated with hippocampal CVR_CO2_ only in women (model’s F_3,193_=5.01, p=0.002).

In subjects without diabetes (n=301) BMI was only marginally associated with hippocampal CVR_CO2_ (model’s F_1,300_=2.6, p=0.10). In fully adjusted analysis *BMI-sex* interaction was significant. Analyses stratified by sex revealed again BMI-hippocampal CVR_CO2_ relationship only in women (model’s F_3,190_=5.1, p=0.002).

In subjects without insulin resistance (n=196, F_1.185_=2.1, p=0.15) the relationship between BMI and hippocampal CVR_CO2_ did not reach significance level.

**Supplemental Table 7.** Linear regression models predicting hippocampal CVR_CO2_  in the entire group, and among women and men separately, using CVR_CO2_  values after taking outliers 2 standard deviations outside the mean.

| **Variable** | **Unstandardized B** | **Standardized β** | **p-value** | **95% CI for B** |
| --- | --- | --- | --- | --- |
| **Entire group** | | | | |
| BMI | -0.054 | -0.10 | 0.08 | -0.115, 0.007 |
|  | | | | |
| age | -0.05 | -0.13 | **0.03** | -0.10, -0.007 |
| sex | 3.95 | 0.70 | **0.04** | 0.28, 7.62 |
| hypertension | -0.33 | -0.06 | 0.34 | -0.99, 0.34 |
| BMI | 0.065 | 0.12 | 0.27 | -0.05, 0.18 |
| *BMI*sex* | -0.15 | -0.71 | **0.03** | -0.29, -0.02 |
| **Women** | | | | |
| age | -0.05 | -0.15 | **0.03** | -0.10, -0.004 |
| hypertension | -0.50 | -0.10 | 0.22 | -1.28, 0.29 |
| BMI | -0.08 | -0.17 | **0.02** | -0.15, -0.015 |
| **Men** | | | | |
| age | -0.05 | -0.09 | 0.34 | -0.14, 0.05 |
| hypertension | -0.07 | -0.01 | 0.91 | -1.28, 1.14 |
| BMI | 0.06 | 0.08 | 0.39 | -0.08, 0.19 |

References

1 Boss, A. *et al.* FAIR-TrueFISP imaging of cerebral perfusion in areas of high magnetic susceptibility differences at 1.5 and 3 Tesla. *J Magn Reson. Imaging* **25**, 924-931 (2007).

2 Rusinek, H. *et al.* Cerebral perfusion in insulin resistance and type two diabetes. *Journal of Cerebral Blood Flow & Metabolism* **35(1)**, 95-102 (2015).

3 Buxton, R. B. *et al.* A general kinetic model for quantitative perfusion imaging with arterial spin labeling. *Magn Reson Med* **40**, 383-396 (1998).

4 Buxton, R. B. Quantifying CBF with arterial spin labeling. *Journal of Magnetic Resonance Imaging* **22**, 723-726 (2005).

5 Stanisz, G. J. *et al.* T1, T2 relaxation and magnetization transfer in tissue at 3T. *Magnetic Resonance in Medicine* **54**, 507-512 (2005).

6 Rusinek, H. *et al.* Hippocampal blood flow in normal aging measured with arterial spin labeling at 3T. *Magnetic Resonance in Medicine* **65**, 128-137 (2011).

7 Glodzik, L. *et al.* Different Relationship Between Systolic Blood Pressure and Cerebral Perfusion in Subjects With and Without Hypertension. *Hypertension.* **73**, 197-205 (2019). <https://doi.org:10.1161/HYPERTENSIONAHA.118.11233>
